# Evaluating the Potential of an Oral-Based Bioguard to Estimate Heart Rate Using Photoplethysmography

**DOI:** 10.3390/bios13050533

**Published:** 2023-05-10

**Authors:** Leonardo de Almeida e Bueno, Victoria C. Walls, Jeroen H. M. Bergmann

**Affiliations:** 1 Department of Engineering Science, University of Oxford, Oxford OX1 3PJ, UK; leonardo.dealmeidaebueno@eng.ox.ac.uk; 2 TCC-CASEMIX, Kestrel Lodge Upper Hexgreave, Farnsfield, Newark NG22 8LS, UK; victoria.walls@tcc-casemix.co.uk

**Keywords:** athlete performance, physiological assessment, biosensors, in-body wearables, physiological measurement, sensor design

## Abstract

The reliable monitoring of heart rate during intense exercise is imperative to effectively manage training loads while providing insights from a healthcare perspective. However, current technologies perform poorly in contact sports settings. This study aims to evaluate the best approach for heart rate tracking using photoplethysmography sensors embedded into an instrumented mouthguard (iMG). Seven adults wore iMGs and a reference heart rate monitor. Several sensor placements, light sources and signal intensities were explored for the iMG. A novel metric related to the positioning of the sensor in the gum was introduced. The error between the iMG heart rate and the reference data was assessed to obtain insights into the effect of specific iMG configurations on measurement errors. Signal intensity was found to be the most important variable for error prediction, followed by the sensor light source, sensor placement and positioning. A generalized linear model combining an infrared light source, at an intensity of 5.08 mA, and a frontal placement high in the gum area resulted in a heart rate minimum error of 16.33%. This research shows promising preliminary results for the use of oral-based heart rate monitoring, but highlights the need for the careful consideration of sensor configurations within these systems.

## 1. Introduction

Heart rate is a vital sign and an important metric in the (initial) assessment of patients. It allows healthcare providers to better understand the physiologic and pathologic processes of an individual [1]. Heart rate is also a useful indicator of physical exertion and captures essential aspects of individuals’ physiological response to exercise. Studies have observed a linear relationship between heart rate and oxygen consumption (VO_2_) under several submaximal conditions [2,3]. Consequently, portable heart rate monitors have become a frequently used tool to estimate exercise intensity, especially when combined with kinematic analysis to determine the physiologic response and metabolic demand experienced in different sports, such as basketball, rugby and football [4,5].

Heart rate monitors use a variety of technologies, ranging from chest-mounted bioelectrical transducers to wrist- or finger-mounted optical sensor devices. One of the main benefits of a wrist strap or an arm strap device is the comfort to the user [6]. However, some of these devices have been demonstrated to underestimate the beats per minute during exercise [6]. Every wrist-worn tracking device performs differently depending on the situation it is used in (e.g., at rest, high-intensity exercise) [7]. A trend has been observed that, at higher heart rates, the measurement error increases, making them less useful in a sporting context, especially during activities that require frequent arm movement, which leads to greater motion artifacts [7,8]. Optical sensors based on photoplethysmography (PPG) have a low signal-to-noise ratio for darker skin tones, thicker or denser tissue and the degree of hydration. This dampens the signal resolution [9]. Current studies in sports medicine indicate that chest strap devices still provide a better level of accuracy at higher heart rates. They are also less susceptible to the aforementioned motion artifacts [7]. Nonetheless, it should be noted that accuracy and precision vary widely between devices.

The field of wearable heart rate monitors is continually evolving, with the public also becoming more interested in monitoring their vitals during daily life. There are many devices publicly available, as well as new technologies in development, for use specifically in contact sports. Several studies have been carried out to develop alternative platforms for monitoring athletes. Such platforms include an epidermal ECG-based sensor, which is similar in appearance to a standard adhesive bandage, applied to the chest [10]. Another novel platform for monitoring heart rate is a garment-based device. This device is specifically designed for use in rugby, and considers the obstruction of the athlete’s performance and measurement during vigorous movements of the body. This wearable system uses ECG, with the electrodes placed on the flank to avoid damage during collisions [11].

Motion artifacts are of particular importance in contact sports, where collisions and general contact will cause the monitoring device to move from its optimum position. These collisions could also damage the heart rate monitoring device. More importantly, devices that are damaged can create a hazard for the players and, therefore, care should be taken to introduce extra equipment in contact sports just for the monitoring device. Therefore, accessories in contact sports, such as watches, are not allowed to be worn during games or training. Even compact and accurate electrocardiogram-based (ECG) devices are not commonly worn during events in which contact with other players or the environment might occur. At the moment, there is a need to generate new solutions that allow for accurate heart rate monitoring during contact sports.

One potential solution to overcome the limitations to the measurement of heart rate in contact sports is to integrate the device into mouthguards. This is already a piece of required protective equipment that is widely used in contact sports. Mouthguards have a lower displacement than helmets and other pieces of sports safety equipment and have been shown to have a displacement under 1 mm during head impact [12]. Mouthguards can also safely accommodate LEDs and other electronic components [13]. In this context, the primary objective of this study was to determine which system configuration generated the lowest error in heart rate estimation from photoplethysmography. The secondary purpose was to identify what the error was in measuring the heart rate from a PPG sensor embedded into a mouthguard.

## 2. Materials and Methods

### 2.1. Participants

For this study, data were collected from seven healthy adult volunteers (4 women and 3 men). The participants had an age range of 21–40 years. All the participants were informed of the general purpose of this study and gave their written consent. The experimental protocols were approved by the Research Ethics Committee of the University of Oxford (R70833/RE001). Our participants were self-declared healthy.

### 2.2. Instrumentation

**Figure 1:**
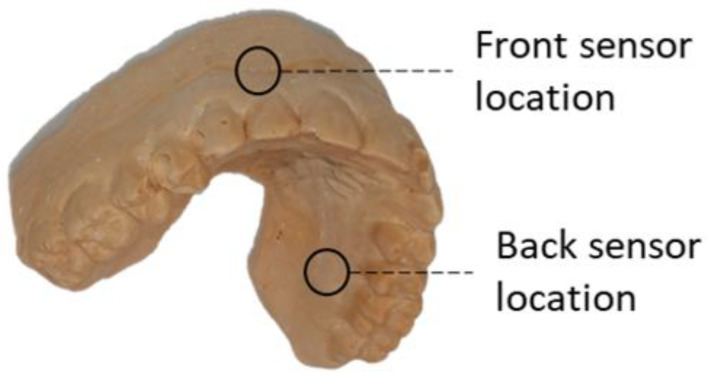
Point of placement for the front and back photoplethysmography sensors. The front sensor was located at the superior alveolar process above the right maxillary lateral incisor to observe blood flow from the middle superior alveolar artery. The back sensor was located at the palate behind the left upper first molar to measure blood flow at the arterioles of the greater palatine artery.

**Figure 2:**
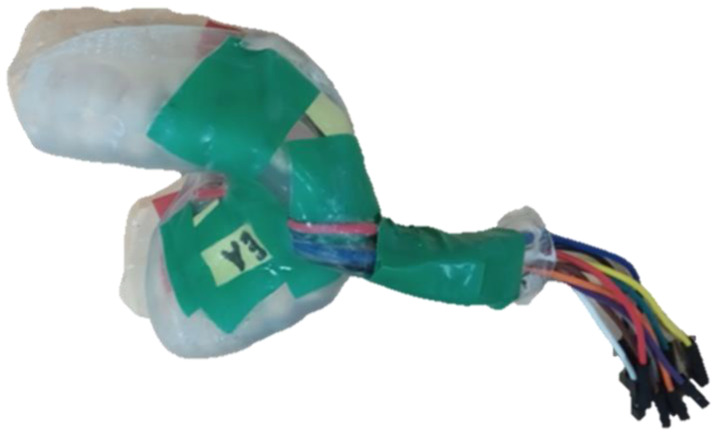
An EVA-encased mouthguard was situated on top of the cast with two MAX30102 sensors embedded. A cable was used to connect it to the MAX30102ACCEVKIT Evaluation System. Green electric tape was used to keep the wires in place during the forming process, while a yellow pH-sensitive comparator paper strip was positioned along the wires to detect potential water ingress.

The oral monitoring device, based on the device used in [14], consisted of a purpose-built custom-made mouthguard designed to measure PPG. It was vacuum formed with ethylene-vinyl acetate (EVA). A maxillary vinyl polysiloxane impression (R&S Turboflex) was taken using a disposable perforated plastic impression (Medibase) and cast in a vacuum-mixed Type 4 dental stone (Singletypo4). One 4.00 mm layer of EVA (Pro-form) was then applied to the working cast with a pressure-moulding machine (Kezham XG-E01). The formed thickness varied about 1–2 mm. The applied sheet was trimmed, leaving a 2–3 mm margin along the gingival margins of the teeth and the posterior borders. Two MAXREFDES117# (Analog Devices, Wilmington, MA, USA) containing MAX30102 (Analog Devices, Wilmington, MA, USA) pulse oximeter devices, with both infrared and red LEDs at 880 and 660 nm, respectively, were secured on the first layer of the EVA by carefully heating the EVA and pressing the sensors into the desired locations. For this study, the sensor board MAXREFDES117# was connected to the MAX30102ACCEVKIT (Analog Devices, Wilmington, MA, USA), which included the USBOSMB microcontroller and the support software. The microcontroller was then connected to a computer using a USB cable which powered the sensor and recorded the data using the MAX30102 Evaluation kit support software. The MAXREFDES117# reference board is smaller than the daughterboard of the MAX30102ACCEVKIT and allowed us to make custom mouthguards for each participant that were all compatible with the same MAX30102ACCEVKIT motherboard. The resulting instrumentation had larger cables coming out of the mouth, but provided a better fit on the gum area, which improved the positioning of the sensors and was more easily reproducible among the participants. Since blood flow variations are more easily observed in arterial blood flow [15], the major arteries and arterioles in the upper mouth were of key consideration. The greater palatine artery (GPA), which is located on the palatal side of the upper mouth and follows the arch of the mouth [16], was of particular interest. Due to the shape of the mouthguard, it was not possible to place the sensor in such a way as to directly take measurements from the GPA, so we placed the sensor on the palatal surface of the upper alveolar process to measure the GPA’s superficial arterioles. One PPG sensor was located at the palatal surface of the upper alveolar process at the right first molar, at least 1 mm above the gumline. Since placing the sensor on the palate is less practical, sites on the buccal side of the suitable teeth should be investigated. One study previously investigated possible positions on both the upper and lower jaw [17]. The positions that provided the best signals in this study were the mental foramen on the lower jaw, through which the mental vessels emerge. However, in the upper mouth signals were still obtained from the gingival area above the maxillary lateral incisor. On the labial side above the incisors, some blood vessels deriving from the middle superior alveolar artery could be measured. For this reason, the second PPG sensor was placed at the superior alveolar process above the right maxillary incisor at least 1 mm above the gumline (Figure 1). Figure 2 shows an example of the mouthguard used for the data capture. A yellow pH-sensitive comparator paper strip was applied to indicate whether water entered into the layer that contained the electronics. The wires from the oral-based components (total length approx. 25 cm) were positioned to exit the mouth from the front, in order to minimise occlusal interferences. They were encased in EVA until they were far enough from the oral cavity. This reduced the chance of saliva affecting the electronics and also kept the wires rigid. Green electric tape was used to keep the wires and electronics in place during the forming process. Sensors were positioned on opposite sides of the mouth to limit the interference between the LEDs of each. A second layer of 1.2 mm EVA was applied once the PPG sensors were in place, which then bonded to the first layer. The excess EVA was trimmed following to the outline of the previously applied layer. The edges were smoothed and rounded with a heated wax knife to improve comfort when being worn intra-orally (Figure 2).

A Polar H10 heart rate monitor (Polar Electro) worn on the chest was used as the gold standard [18]. Heart rate data from the Polar H10 were recorded using the Polar Beat android application version 3.5.5. Reference devices sampled the heart rate data at 1 Hz.

### 2.3. Experimental Protocol

#### 2.3.1. In Vivo Testing

The participants were asked to wear the instrumented mouthguard and the Polar H10 heart rate monitor while seated on a chair and breathing normally. Using the MAX30102ACCEVKIT, both the red and infrared lights of the MAX30102 pulse oximeters inside of the mouthguards were sampled at 200 Hz, without averaging, a pulse width of 400 µs and an ADC full-scale range of 8192 nA. The sample rate was chosen for compatibility with the commonly used data processing methods for the tracking of heart rate from PPG signals affected by motion artifacts [19]. Light-emitting diode (LED) currents were set at six different peak light intensities: 5.08 mA, 12.5 mA, 18.75 mA, 25 mA, 31.25 mA and 37.5 mA. The Polar H10 and the instrumented mouthguard were activated simultaneously, and the time was registered to ensure data alignment. The Polar H10 is regarded as an appropriate gold standard for heart rate measurement in sports applications [20]. Thirty minutes of data were recorded for each sensor at each LED intensity and totalled 6 h of data for each subject. The data were recorded in 10 min epochs after which the participants were allowed to remove the device before returning for the next 10 min data acquisition session. The breaks between data collection sessions allowed the participants to ingest accumulated saliva, drink water, walk, rest or even continue the data acquisition at a later occasion. As a consequence, the effects of involuntary movements resulting from salivation were mitigated as much as possible and equalized throughout the data.

#### 2.3.2. In Vitro Testing

**Figure 3:**
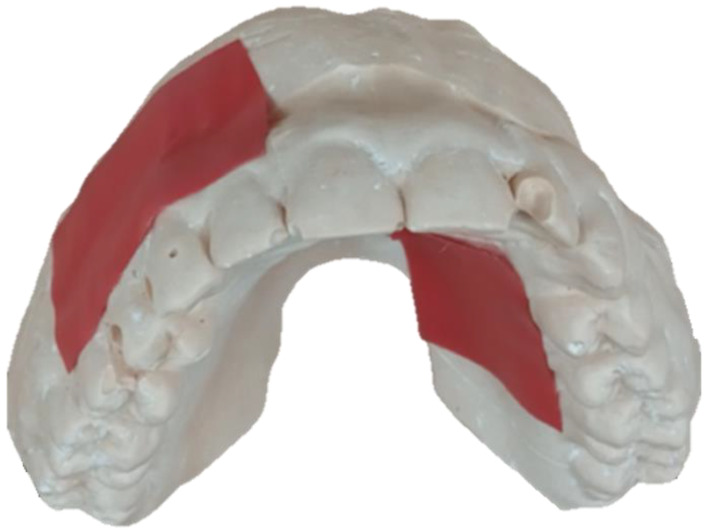
A dental cast with red tape marking the area beyond the gumline likely to contain a high density of arterioles.

To quantify the manufacturing variability, for each participant the PPG-baseline cast reflectance was measured by positioning the participants’ mouthguards on their original casts and using the MAX30102ACCEVKIT to record 20 s of the cast reflection from the MAX30102 pulse oximeters inside of the mouthguards. Both the red and infrared lights of the sensors were sampled at 200 Hz, without averaging, a pulse width of 400 µs, an ADC full-scale range of 8192 nA and peak LED currents of 5.08 mA. Subsequently, red electrical tape was placed on the cast marking the area above the gum line, where the blood vessels are expected to be (Figure 3). Another measurement of the MAX30102 pulse oximeters inside the mouthguards on the taped cast was performed using the same configuration of the baseline cast reflection.

**Figure 4:**
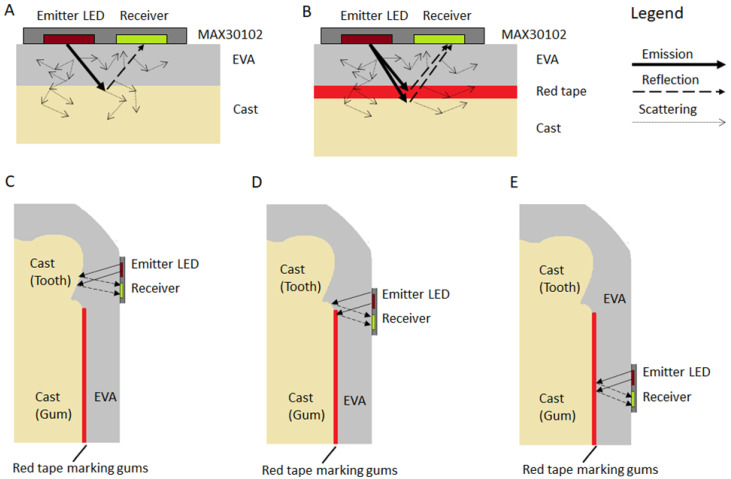
Model of light propagation in the mouthguard. (**A**) Baseline cast without tape. Light is expected to scatter both in the EVA and in the cast material. (**B**) Taped cast. The red tape should change the reflection of the red and infrared light in comparison to the bare cast. (**C**) The PPG positioned closer to the teeth area should have most of the light reflected by the cast material. (**D**) The PPG positioned in between the teeth area and the gum should reflect light affected by an area of only cast material and an area covered by the red tape. (**E**) The PPG is positioned completely in the gum area and most of the light reflection should be affected by the taped cast.

As represented in Figure 4, due to the colour of the cast material a different reflection was expected from the casts with and without red tape, resulting in an observable intensity change in the signal captured by the receiver when compared to the base cast. Figure 4C–E illustrates how different sensor positions can affect the reflected signal. If the sensor is placed next to the teeth area, the reflected signal will be similar to just reading the baseline cast reflection. The more the signal is moved to the gum area, the more the reflected signal is affected by the tape. For this investigation, the reflection of the taped area needed to be considerably different from the baseline cast. For this reason, red tape was used. By dividing the average of the taped measurement by the average of the baseline cast reflectance, we deduced a metric of sensor misplacement. If the sensors were poorly placed, being closer to the teeth, the reflection ratio would tend to 1, indicating that the PPG sensors were measuring more of the teeth (cast reflection) and fewer blood vessels. This metric, called Gum Reflection, is hypothesised to impact sensor accuracy, as previous studies have demonstrated that pulse oximetry measurements from the teeth are poorly correlated to pulse oximetry from the fingertips, due to the limited blood vessels inside of the teeth [21]. Four readings of Gum Reflectance, representing each combination of position and light source (front red, front infrared, back red, back infrared), were obtained for each participant.

### 2.4. Signal Processing

The primary goal of the data analysis was to identify the hardware configuration that resulted in the lowest error. The signal processing used basic methods commonly applied to the extraction of heart rate data from photoplethysmography (PPG) signals while avoiding the masking errors caused by the different hardware configurations.

**Figure 5:**
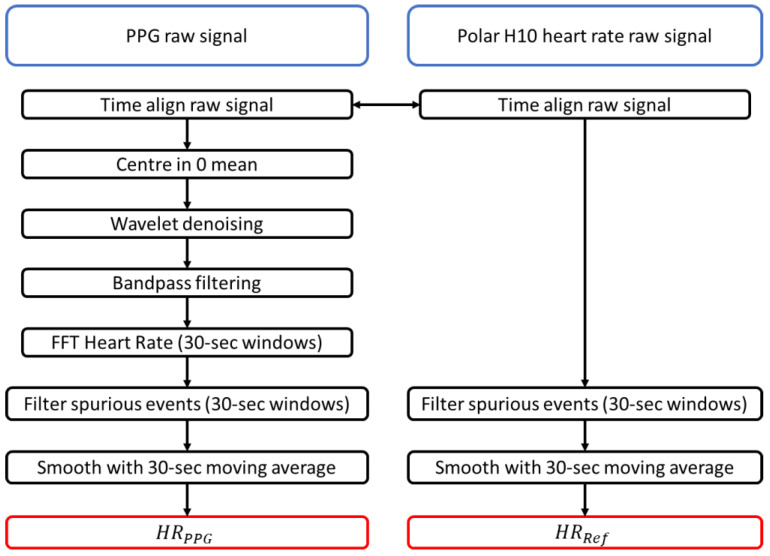
Flowchart of signal data processing.

All the data processing was performed after the experiments. The PPG and reference data were time aligned to allow for synchronous processing. The PPG data were denoised using a wavelet denoising algorithm based on the discrete wavelet transform (DWT) with the db4 mother wavelet [22]. The wavelet threshold was the soft rigsure shown to improve the denoising performance [23]. The denoised signal was then normalized and filtered using a 4th-order zero-phase bandpass filter between 0.5 Hz and 4 Hz, as frequently performed on PPG signals affected by motion artifacts [19]. The resultant signal was used to calculate the heart rate using the Fast Fourier Transform (FFT) algorithm described in [24]. This algorithm estimates the heart rate from the main frequency component in a window of the signal. To observe the effect of short-term heart rate variability, the heart rate was calculated using moving windows from the previous 30-s data. The windows had 1-s steps to allow for a direct comparison with the reference device. Readings resulting from spurious events were filtered out from both the reference signal and the calculated heart rate using the localized mean and standard deviation over 30-s windows, a method commonly used in sports science [25]. The first calculated window was discarded as its data included misreadings from when the sensors were activated. Finally, the calculated heart rate and the reference HR data were both smoothed using a moving average with the same 30-s window size. The outputted data from the processing were ${HR}_{PPG}$, the heart rate was calculated from the PPG signal and ${HR}_{Ref}$ was the heart rate from the reference Polar H10. The processing steps of the raw and the reference signals are illustrated in Figure 5. The data were processed using Matlab 2019b (Mathworks, Natick, MA, USA).

### 2.5. Statistical Analysis

A generalised linear model (GLM) was built using a stepwise regression method performed on the denoised heart rate data. The sensor position (front or back), light source (red or infrared) and peak light intensity (5.08 mA, 12.5 mA, 18.75 mA, 25 mA, 31.25 mA or 37.5 mA) were assigned as categorical predictors and gum reflection was assigned as a continuous predictor. The prediction terms were added to or removed from the model based on the model deviance. A normal distribution was assumed for the output and a *p*-value < 0.05 was considered statistically significant. This model was then applied to understand how each predictor contributed to the reading of the absolute percentage error measured as:

(1) $$\left|\frac{{HR}_{Ref}-{HR}_{PPG}}{{HR}_{Ref}}\right|\;*\;100\%$$

where ${HR}_{Ref}$ is the heart rate measured using the Polar H10 and ${HR}_{PPG}$ is the calculated heart rate. Errors were calculated point-by-point for each participant.

Considering that the error output was potentially non-parametric, Kruskal–Wallis tests were also conducted to examine the differences in output error according to the hardware configuration and further confirm any findings of the GLM. QQ-plots and histograms were used to visually inspect whether normality could be considered. Non-parametric tests were performed if normality could not be assumed. The null hypothesis was that there was a significant difference in the errors resulting from sensor position (front or back), light source (red or infrared) and peak light intensity (5.08 mA, 12.5 mA, 18.75 mA, 25 mA, 31.25 mA or 37.5 mA). If the output errors were significantly different in the independent groups, a multiple comparison test was performed in a post hoc manner to identify the specific differences among the groups. A *p*-value < 0.05 was considered statistically significant.

## 3. Results

The average heart rate of all the participants during the experiments, as measured using the Polar H10, was 71.92 (SD ± 9.78) beats per minute (bpm).

### 3.1. Heart Rate Calculation without Wavelet Denoising

**Figure 6:**
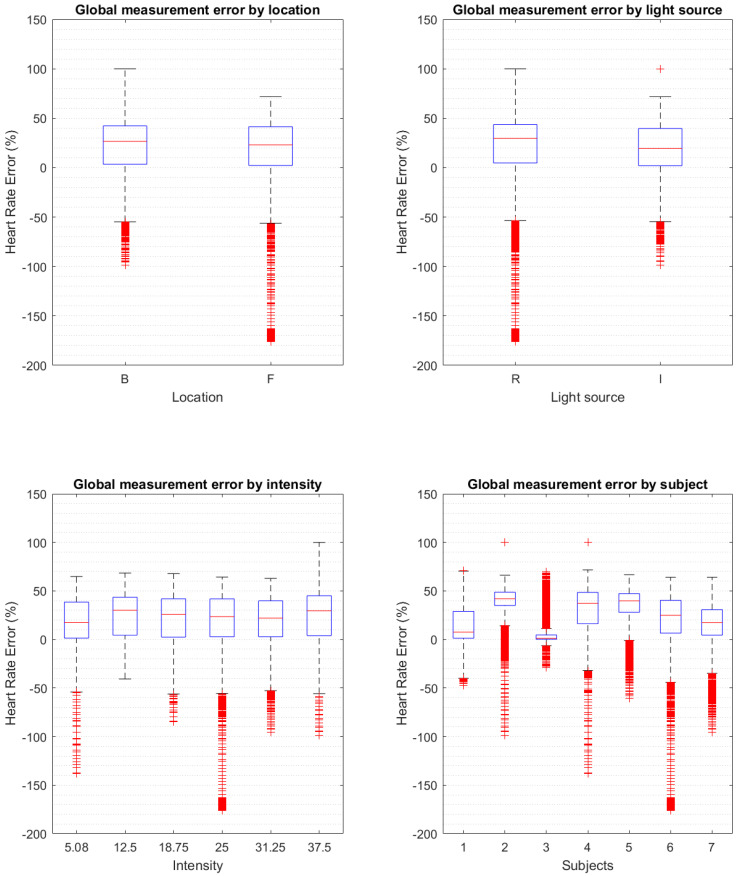
Boxplots of the error of heart rate calculation from the PPG without wavelet denoising separated by error source predictors. The subjects’ boxplot is displayed to expose the difference in the manufactured mouthguards that was assessed using the gum reflection metric. The central lines of the boxplots are the median, the whiskers are the upper and lower quartiles and the outliers are points 1.5 times above the upper and lower quartiles.

**Figure 7:**
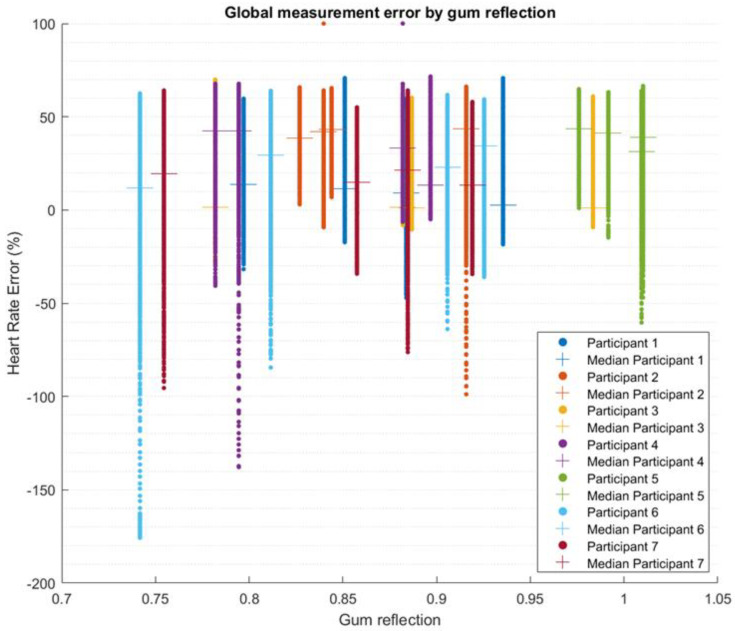
Scatter plot of the error of heart rate calculation from the PPG without wavelet denoising separated by gum reflection metric. Each participant had four gum reflection metrics that consisted of front red, front infrared, back red and back infrared. All data are shown in this plot.

The data points without wavelet denoising from all the tests associated with each error score were binned together to form the boxplots of the predictors against percentage error (Figure 6). A scatter plot of the error against the gum reflection is presented in Figure 7.

### 3.2. Signal Processing with Wavelet Denoising

**Figure 8:**
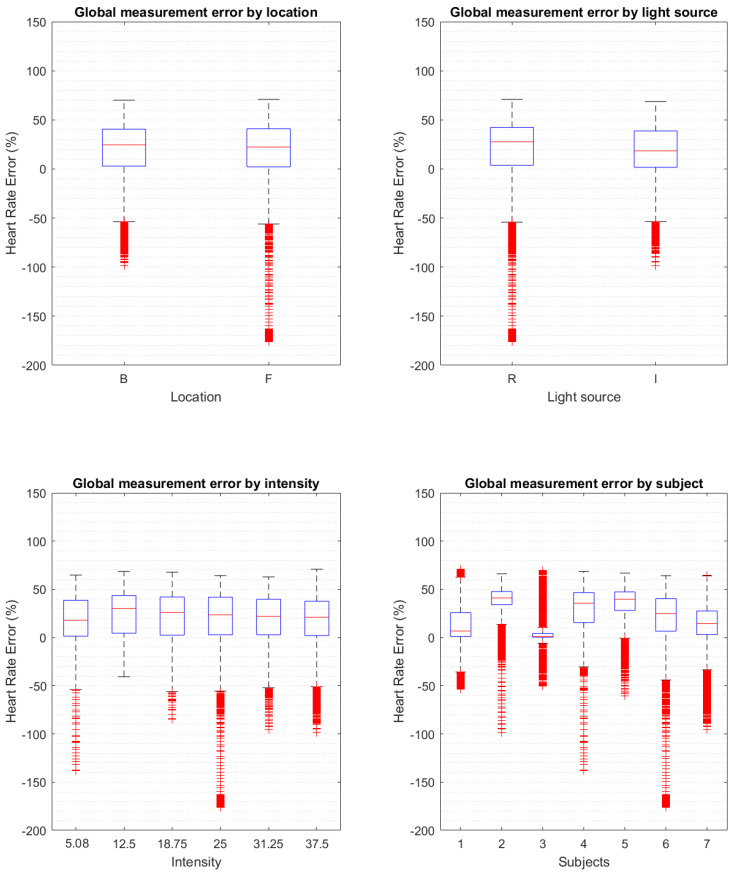
Boxplots of the error of the heart rate calculation from the PPG separated by error source predictors. The subjects’ boxplot is displayed to expose the difference in the manufactured mouthguards that was assessed using the gum reflection metric. The central lines of the boxplots are the median, the whiskers are the upper and lower quartiles and the outliers are points 1.5 times above the upper and lower quartiles.

**Figure 9:**
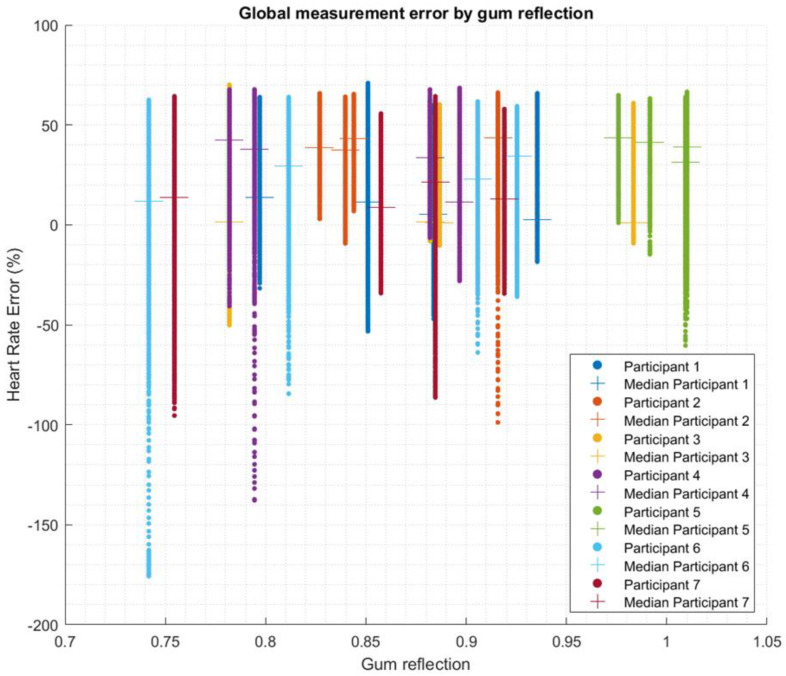
Scatter plot of the error of the heart rate calculation from the PPG separated by the gum reflection metric. Each participant had four gum reflection metrics that consisted of front red, front infrared, back red and back infrared. All the data are shown in this plot.

**Figure 10:**
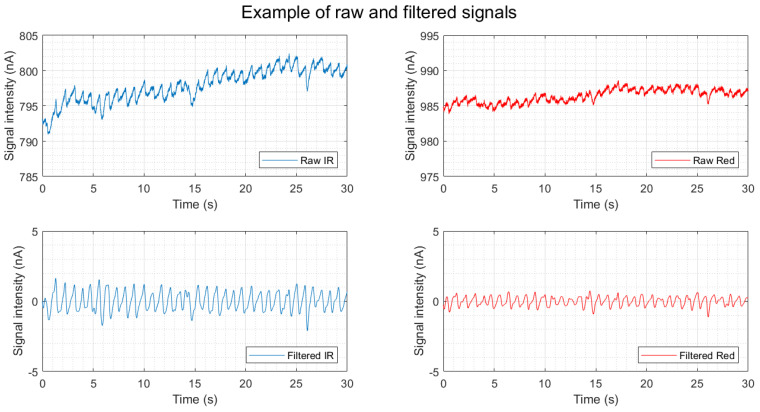
Example of raw (top figures) and filtered (bottom figures) PPG signals at a light intensity of 5.08 mA. The left side shows the data for the infrared (IR) and the right side for the red LED.

All the data points from all the tests associated with each error score were binned together to form the boxplots of predictors against percentage error (Figure 8). A scatter plot of the error against the gum reflection is presented in Figure 9. An example of a raw and filtered signal can be found in Figure 10.

A stepwise regression analysis was performed to build a GLM and assess how much the predictors accounted for the percentage error in the calculated heart rate. The final univariate GLM consisted of the determinants and their interactions that best predicted the error. It included the light intensity, source, position and gum reflection. A light intensity of 5.08 mA was predicted to generate the lowest error, while an intensity of 12.5 mA resulted in the highest error prediction. The difference between the minimal and maximum prediction errors due to light intensity was 5.58%. The infrared light source had the lowest error prediction. A difference of 5.40% was found between the minimum and maximum for the different light sources. A front sensor placement resulted in the lowest error prediction, with a difference of 1.12% between the errors related to the sensor location. A low gum reflection resulted in a low error prediction (gum reflection error difference was 3.29%). The sensor placement was not included in the final GLM.

The lowest prediction error was obtained for the model containing a light intensity of 5.08, an infrared light source, front placement and a gum reflection of 0.74. This yielded a 16.33% error prediction with a 95% CI from 15.75 to 16.90%.

Kruskal–Wallis tests were conducted to examine the differences in output error according to the hardware configuration (Table 1). Significant differences were found among all the hardware configurations (chi-square = 6297.19, *p*-value = 0, df = 9). The use of infrared light was found to result in lower errors than red light (*p*-value < 0.001); front placement was found to result in lower errors than back placements (*p*-value < 0.001). A light intensity of 5.08 mA resulted in the lowest errors while an intensity of 12.5 mA resulted in the highest errors (*p*-value < 0.001).

## 4. Discussion

This study compared the impact of hardware design strategies for the measurement of heart rate using photoplethysmographic sensors embedded in the oral cavity. A novel metric, gum reflection, was introduced to assess the sensor’s proximity to the blood vessels, under the assumption that proximity to the teeth would result in a higher reflection of the dental material. The impact of the sensor location, light source, light intensity and gum reflection on the error difference between a reference ECG sensor and the mouthguard PPG was estimated using a generalized linear model approach. The lowest error was achieved with a light intensity of 5.08 mA, an infrared light source, a front placement of the sensor and a gum reflection of 0.74. This approach resulted in a minimum error of 16.33%. Since these errors can influence the parametric assumptions of the GLM, the hardware configurations were also assessed using a Kruskal–Wallis test followed by post hoc tests. The outcome, that a light intensity of 5.08 mA, an infrared light source and a front placement of the sensor resulted in the lowest errors, was further confirmed with these tests.

While the minimum error was achieved with the lowest intensity (5.08 mA), an intensity of 12.5 mA resulted in the largest error. Higher light intensities resulted in intermediate errors, with intensities of 18.5 mA and 25 mA not showing any statistically different outputs. These results are in line with the literature and can be explained by the different contributions of the light to the direct current (DC) and pulse amplitude (AC) components of the photoplethysmographic signal. The AC component of the signal reflects the change in blood flow and is used to detect heart rate. The DC component of light includes scattering and reflectance in body tissues. Lower light intensities were shown to have a better signal-to-noise ratio, while increases in light intensity contributed to increases in the DC components but an increase, followed by a decrease, in the AC components [26]. In addition, the mucous membranes found in the gums do not have the keratinizing layer observed in the skin and may require a lower signal intensity to reach the blood vessels. The high light intensities also resulted in the saturation of some subjects. That happened almost exclusively at the intensities of 31.25 mA and 37.5 mA, although there was variation among participants. This was reflected by the large positive errors found when no wavelet denoising was applied. Saturation at high intensities was more often observed towards the end of the data collection epochs, when possibly a build-up of saliva could have affected the sensors. Further studies need to be performed to investigate these factors. The wavelet denoising was capable of filtering out most of these errors.

Sensor placement above the maxillary lateral incisor or along the grand palatal artery mildly contributed to the errors in measurement. However, the placement effect on the heart rate was statistically significant. This result needs to be investigated further and among the hypotheses, it is imperative to explore the possible lower salivation with a labial placement when compared to a palatal placement. The front sensor was not directly facing the labial glands, while the back sensor was positioned directly at the palatine glands and was also close to the major salivary gland ducts. This, in combination with the shape of the mouthguard, may have allowed for a larger build-up of saliva in the back placement. Another aspect to be investigated is the difference between the mucous membrane in the palate and the labia. The palatal surface has a thicker membrane for chewing whereas the labial side is covered in a thinner membrane. This factor may explain the differences in the readings between the front and back, although it does not explain the saturation of the sensors towards the end of the measuring epochs when saliva accumulation is expected. It might be that in larger sample sizes different effects will be observed, but based on these preliminary results the sensor should be placed in the front position at the labial side of the maxillary lateral incisor.

The gum reflection parameter indicates that the manufacturing process should consider the proximity of the optical sensor to the teeth. Gum reflection values closer to one indicate that the PPG sensor is reading light signals reflected by the teeth, while lower values indicate that the optical sensor is reading light reflected by the gum tissue. Research has demonstrated that although dental pulp could be used to determine pulp vitality, the tooth oxygen saturation does not correlate with finger PPG readings because of the teeth’s material and health [21]. The gum reflection scatter plots illustrate the impact of the placement of the sensors. Any variability in sensor placement during the manufacturing process should be considered, as this could change the location relative to the gum. However, the fit of the mouthguard, the signal dispersion in the EVA material, the movement of the sensors inside the mouthguard encasement and the accumulation of saliva in front of the sensors are among the variables that need to be further investigated to identify other potential factors that affect measurement errors.

The infrared light source resulted in lower errors compared to the red light source. Infrared light has longer wavelengths and better penetrates the tissue and, for this reason, it is often used in photoplethysmography. Our results indicate that once embedded in the mouthguard, the longer wavelength was probably less affected by the mouthguard’s material, which would scatter the signal and increase the distance to the tissues and vessels. Indeed, when looking at Figure 10, with examples of raw and filtered signals, we can notice that although the red light resulted in a higher DC component than the infrared light, the AC component was smaller and the filtered signal had a lower signal-to-noise ratio. For the application to a wearable device with power constraints, such as the mouthguard, the longer wavelength has the added benefit of being more power efficient than shorter wavelengths, such as green light. However, this study did not control for the direct impact of motion artifacts, salivation or light scattering on the mouthguard material; once these factors are more carefully analysed a shorter wavelength of light may be demonstrated to mitigate the errors.

The processing methods used were chosen to initially minimize bias and allow for the investigation of the sources of error in the system. This approach avoided the fully masking issues derived from the controlled system configurations, but still had some drawbacks. The FFT heart rate algorithm used was easily implementable and computationally efficient; however, the results indicate that the algorithm failed to estimate the heart rate within a reasonable interval in some situations, even when using basic methods for filtering and denoising the data. This was probably due to the artifacts caused by swallowing, chewing on the mouthguard or talking. All of these actions may affect the sensor placement. The accumulation of saliva may also account as a form of error, especially at high light intensities, although how this impacts the signal is still unclear from the analysis performed. A drawback of the FFT method is that significant motion artifact could result in the erroneous calculation of the heart rate. The FFT is also based on the assumption that the signal is stationary, which is not the case for PPG signals, and so errors can arise.

A more robust algorithm might further reduce the identified errors. In this study, we made use of wavelet denoising to mitigate motion and light saturation artifacts. The benefit of this method is that it represents both the time and frequency aspects of the signal, which is important since the signal is time-varying. In [22], the discrete wavelet transform (DWT) with the Daubechies wavelet had the best performance in the estimation of heart rate from the PPG signal. Indeed, when observing the box plots of the errors with and without wavelet denoising we observe that some of the extreme errors were filtered out and the error variance was reduced. This algorithm, however, was not able to filter out all spurious events, indicating that a more robust processing, such as adaptative filtering [19] or a PPG peak detection method [27], needs to be investigated, while manufacturing issues that largely affect the signals also need to be addressed.

The errors identified were significantly above the accepted clinical standard of up to 5%. Since this investigation was primarily focused on identifying the best hardware configuration and this device was being designed for use in sports rather than for medical devices, a greater error could be acceptable, but not ideal. The error was likely from noise which could appear due to age, gender or conditions of the upper mouth [28], as well as the aforementioned salivation, motion artifacts and light scattering through the mouthguard. Similar causes of error are found in other PPG devices and, when compared to a similar ground truth ECG heart rate monitor, such devices display a mean bias of −5.9 bpm [6].

This study had several limitations. Although designed to be used in sports, the current study tested the sensors with the participants at rest, where the heart rate did not vary much. A relatively small sample group was used and an extrapolation of its findings should be taken carefully. From a technical perspective, the use of cables between the lips for 10 min epochs resulted in salivation and consequently some potentially involuntary movements. This factor possibly contributed to some of the errors observed in our study. Moreover, as mentioned previously, the build-up in saliva over time might have contributed to saturation in the high-light intensity configurations, potentially increasing the errors.

Nonetheless, following the best practices for developing medical technologies, the preliminary testing and verification of subsystems, the technological assumptions and the integration of all components are paramount for the successful validation and application of systems [29]. This study focused on the first phase of the development process. For the IMG-enabled heart rate measurements, further investigations are needed to quantify how heart rate variability, light scattering, motion artifacts, and salivation affect the sensor performance before field testing commences.

There appears to be sufficient evidence that a device such as this is a viable solution for measuring heart rate from the oral cavity. The results indicate that with the appropriate system configuration and signal processing, the performance is comparable to other devices which are used for physiological monitoring in sports, and so its potential to contribute to enabling the safer practice of contact sports is promising.

## 5. Conclusions

This study determined the main factors impacting heart rate measurement errors taken from mouthguards instrumented with PPG sensors using both lab bench and in vivo tests. It identified that sensor placement in reference to the gum, LED light intensity and wavelength significantly affected measurement errors. More importantly, this study provided support for the idea that it might be possible to move the continuous and objective monitoring of physiological signals away from controlled tests and onto the field during a varied range of exercise practices, including contact sports. This will create an opportunity to better understand player welfare and performance under real-world conditions that relate to the specificity of the physical activity performed.

## Figures and Tables

**Table 1:** Kruskal–Wallis mean ranks of hardware configurations.

Hardware	Configuration	Mean Rank
Light Source	Red	${458.87\times 10}^{3}$
	Infrared	${401.20\times 10}^{3}$
Placement	Back	${436.71\times 10}^{3}$
	Front	${423.35\times 10}^{3}$
Intensity	5.08 mA	${390.83\times 10}^{3}$
	12.5 mA	${464.03\times 10}^{3}$
	18.75 mA	${436.04\times 10}^{3}$
	25 mA	${439.31\times 10}^{3}$
	31.25 mA	${419.89\times 10}^{3}$
	37.5 mA	${429.34\times 10}^{3}$

## Data Availability

Not applicable.
